# The Lungs Before and After COVID-19 Pneumonia

**DOI:** 10.4269/ajtmh.20-0357

**Published:** 2020-05-14

**Authors:** Marcello A. Orsi, Giancarlo Oliva, Michaela Cellina

**Affiliations:** Department of Radiology, ASST Fatebenefratelli Sacco, Milan, Italy

A 69-year-old woman was admitted to our Emergency Department with cough and dyspnea. Laboratory investigations showed increased white blood cell count (10,500 per μL), and C-reactive protein levels (124 mg/dL). Chest X-ray (Figure 1A) showed findings consistent with bilateral interstitial pneumonia; COVID-19 was detected in a throat swab sample by RT-PCR.

**Figure 1.**
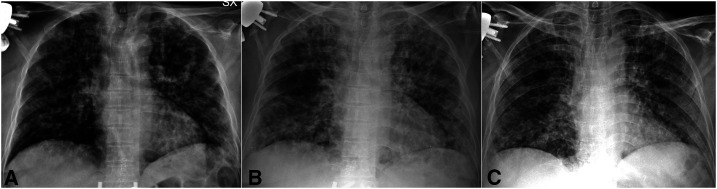
Plain X-rays. (**A**) Chest X-ray on admission showed bilateral pulmonary infiltrates. The distribution pattern is mainly peripheral. (**B**) Chest X-ray on day 14 showed worsening of the radiologic findings with peripheral and central distribution and bilateral evidence of areas of consolidation, particularly in the right lower field. (**C**) Chest X-ray on day 25 showed improvement of the radiological findings, particularly in the upper and middle fields, on both sides.

From day one, the patient suffered from a type I respiratory failure requiring supplemental oxygen through a Venturi mask, FiO_2_ up to 60%. She was treated with lopinavir/ritonavir and hydroxychloroquine; because of the persistent severe conditions (Figure 1B), tocilizumab was added on day 14. Two days later, after improvement in respiratory exchange, the oxygen therapy was suspended. A slow progressive improvement occurred (Figure 1C), and on days 30 and 31, RT-PCR was negative.

On day 32, the patient underwent an unenhanced chest computed tomography (CT), showing diffuse lung architectural distortion, with reticular interstitial pattern, peripheral honeycombing, and bronchial wall thickening.

Images of a previous chest CT performed by the patient a year ago are available, showing no significant abnormalities.

The dramatic pulmonary changes (Figure 2A and B) make us question the possible long-term consequences of COVID-19 pneumonia.

## Figures and Tables

**Figure 2.**
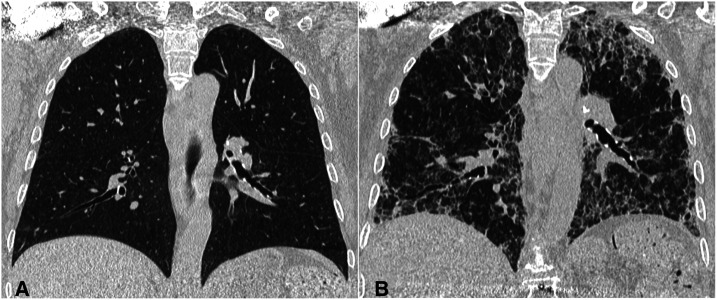
Chest computed tomography coronal reconstructions: (**A**) executed in 2019, showing no abnormalities; (**B**) executed in 2020, after COVID-19 infection. Diffuse lung architectural distortion is visible.

